# A maternally derived complex small supernumerary marker chromosome involving chromosomes 8 and 14: case report and review of the literature

**DOI:** 10.3389/fgene.2024.1331676

**Published:** 2024-02-23

**Authors:** Fatima Ouboukss, Zhour El Amrani, Hicham Bouchahta, Ilham Ratbi, Aziza Sbiti, Thomas Liehr, Abdelaziz Sefiani, Abdelhafid Natiq

**Affiliations:** ^1^ Faculty of Medicine and Pharmacy, Research Team in Genomics and Molecular Epidemiology of Genetic Diseases, Genomics Center of Human Pathologies, University Mohammed V in Rabat, Rabat, Morocco; ^2^ Department of Medical Genetics, National Institute of Health in Rabat, Rabat, Morocco; ^3^ Institute of Human Genetics, Jena University Hospital, Friedrich Schiller University, Jena, Germany

**Keywords:** complex small supernumerary marker chromosome (sSMC), partial trisomy, molecular cytogenetics, clinical features, chromosome 8 and 14

## Abstract

**Introduction:** The majority of small supernumerary marker chromosomes (sSMCs) are derived from one single chromosome. Complex sSMCs, on the other hand, consist of genetic material derived from more than one, normally two chromosomes. Complex sSMCs involving chromosomes 8 and 14 are rarely encountered.

**Case presentation:** We present here a 14-month-old boy born from an unrelated couple. At birth, the baby was hypotonic and had a cleft lip and palate, as well as ocular involvement. Throughout the course of development, the baby experienced feeding difficulties, stunted growth, and delayed psychomotor development. Banding together with molecular cytogenetics revealed a balanced maternal translocation t(8;14)(p22.3;q21)mat, leading due to meiotic 3:1 segregation to a partial trisomy of chromosomes 8 and 14 in the affected boy.

**Discussion/Conclusion:** This report highlights the importance of cytogenetics in diagnosis of rare genetic disorders, with impact on genetic counselling of patients and their families. There are three comparable cases in the literature involving both chromosomes 8 and 14, but with different breakpoints; the complex sSMC derived from chromosomes 8 and 14 in this case, characterized as der(14)t(8;14) (p22.3;q21)mat.

## Introduction

Small supernumerary marker chromosomes (sSMCs) are structurally abnormal chromosomes that are difficult to identify or characterize unambiguously using conventional banding cytogenetics alone. These sSMCs are typically equal in size or smaller than chromosome 20 of the same metaphase spread (Liehr et al., 2004).

sSMCs have been found to be derived from any of the 24 human chromosomes, including both autosomes and gonosomes, with a predominant source being chromosome 15, followed by chromosome 22 (Liehr, 2023). sSMCs have been reported in various populations, with occurrences noted as follows: 0.043% in newborn infants, 0.077% in prenatal diagnosis cases, 0.433% in patients with intellectual disability, and 0.171% in individuals experiencing subfertility (Liehr and Weise, 2007). sSMCs may have different shapes and constitutions, like ring, centric minute and inverted duplication shape. Also, they may be continuous, discontinuous, single, multiple, neocentric, complex or form other rare subgroups as summarized in Liehr 2023. One of the smallest sSMC subgroups is constituted by the so-called complex sSMCs, they contain chromosomal material originating from more than one, normally two chromosomes (Trifonov et al., 2008; Liehr et al., 2013; Liehr, 2023).

The clinical presentation of sSMCs shows significant variability, and they are detected unexpectedly in routine karyotype analyses (Liehr et al., 2010). In our routine chromosome analysis, a 14-month-old boy was found to have an sSMC. R-banding technique showed 47,XY,+mar (ISCN, 2020). His father’s karyotype was 46,XY, while in his mother a balanced reciprocal translocation between chromosomes 8 and 14 was detected.

## Case presentation

**FIGURE 1:**
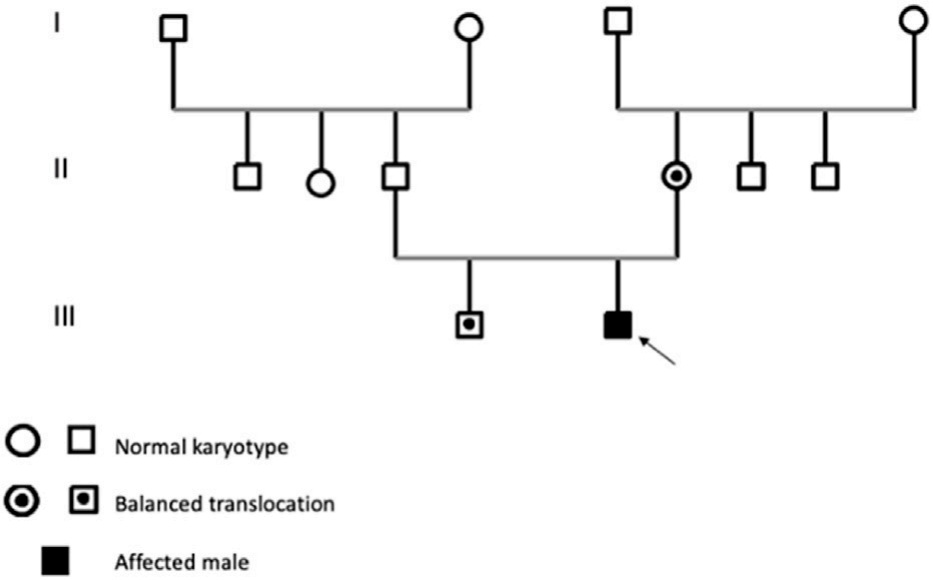
Pedigree balanced translocation in the mother’s family.

A 14-month-old infant, born to unrelated parents, the second child in a two-sibling family, with his older brother in good health is reported (Figure 1). The mother’s pregnancy was uneventful; she was 27 years old at the time of birth, while the father was 34. The patient’s birth weight was 3 kg, with a head circumference 32 cm (P50); he had an APGAR score of 8/10. At clinical evaluation at 14 months of age, the patient showed growth delay in terms of height and weight, delay in psychomotor development, and generalized hypotonia with feeding difficulties. The patient also exhibits dysmorphic features, including microcephaly, craniosynostosis, broad and prominent forehead, hypertelorism, microphthalmia, narrow palpebral fissures, arched eyebrows, flattened nose with wide nostrils, low-set ears, umbilical hernia and clinodactyly. Additionally, he had undergone surgery for a cleft lip and palate. His echocardiography, abdominal ultrasounds and brain MRI were normal.

## Materials and methods

Parents gave informed consent for the genetic analysis, which was performed in accordance with the Declaration of Helsinki protocols and approved by the local institutional review boards. Venous blood (3–5 ml) acquired in a heparinized tube was taken of the patient and his parents for RHG cytogenetic analysis (R-banding of human chromosomes by heat denaturation and Giemsa staining). About 0.4–0.8 ml of peripheral blood was incubated in complete lymphocyte culture medium for 72 h. Metaphases were harvested by adding KaryoMAX™ Colcemid™ solution for 50 min followed by hypotonic KCl (0.075M) treatment for 20 min and fixation using standard 3:1 methanol and acetic acid fixative (Dutrillaux and Couturier, 1981). Thereafter, fluorescence *in situ* hybridization (FISH) assay was performed to characterize the sSMC, it was performed on patient’s and his mother’s metaphases obtained from whole blood cultures. Multi-FISH applying three probes as whole chromosome painting (wcp) probes for chromosomes 8 and 14 (homemade, labelled in Cyanine 5 (yellow) and diethylaminocoumaine (DEAC) (as described in Liehr and Claussen, 2002), and the subtelomeric 8pter in Spectrum Green (D8S504, Abbott/Vysis, Germany) specific to the subtelomeric region 8p23.3. Chromosomes were counterstained by 4′,6-diamidino-2-phenylindole (DAPI). FISH technique was performed according to standard procedures (Liehr, 2002). Image acquisition was done using a Zeiss Axioplan microscope equipped with ISIS software (MetaSystems, Altlussheim, Germany) and 15 metaphases were analyzed.

## Results

**FIGURE 2:**
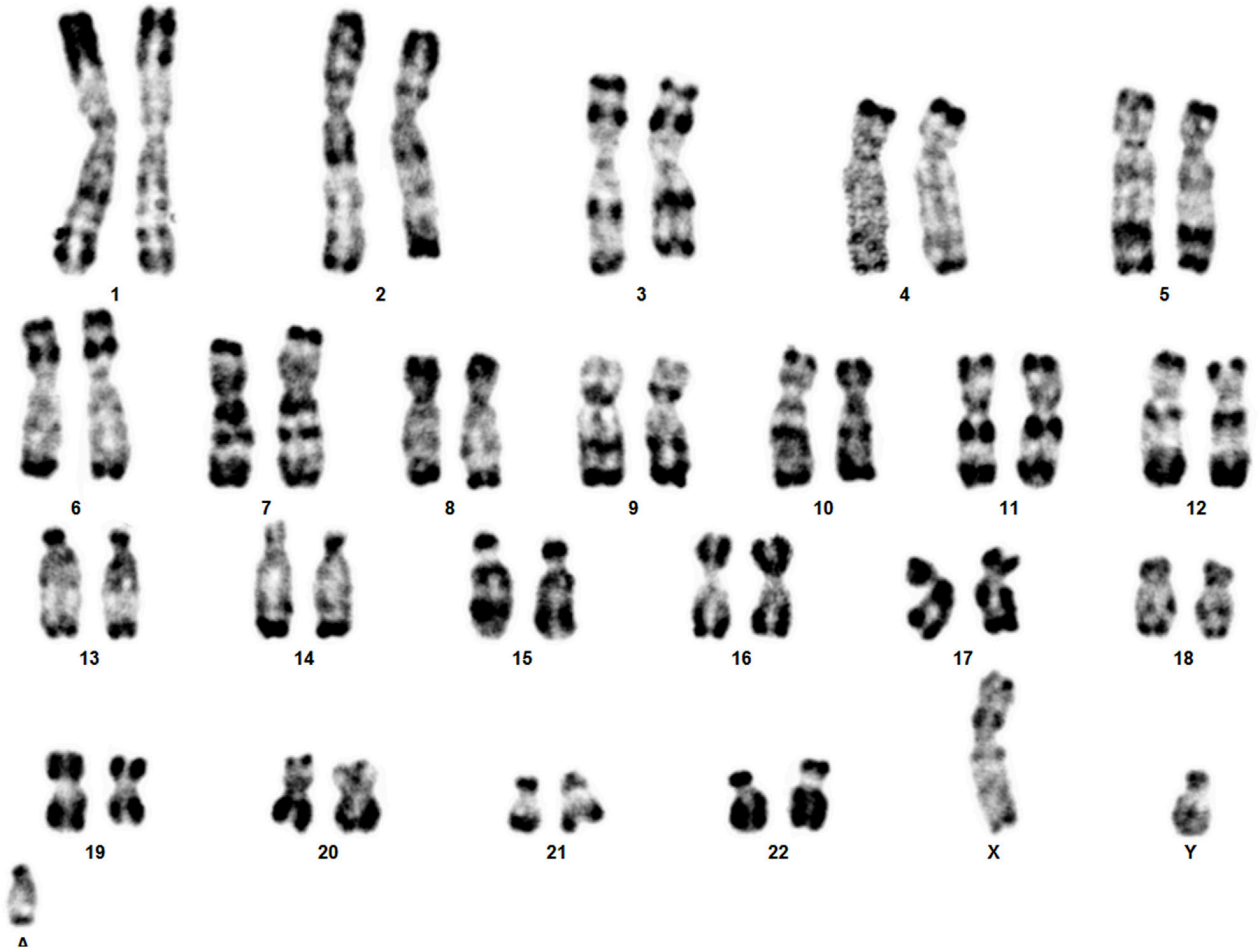
The karyotype of the patient showing sSMC.

**FIGURE 3:**
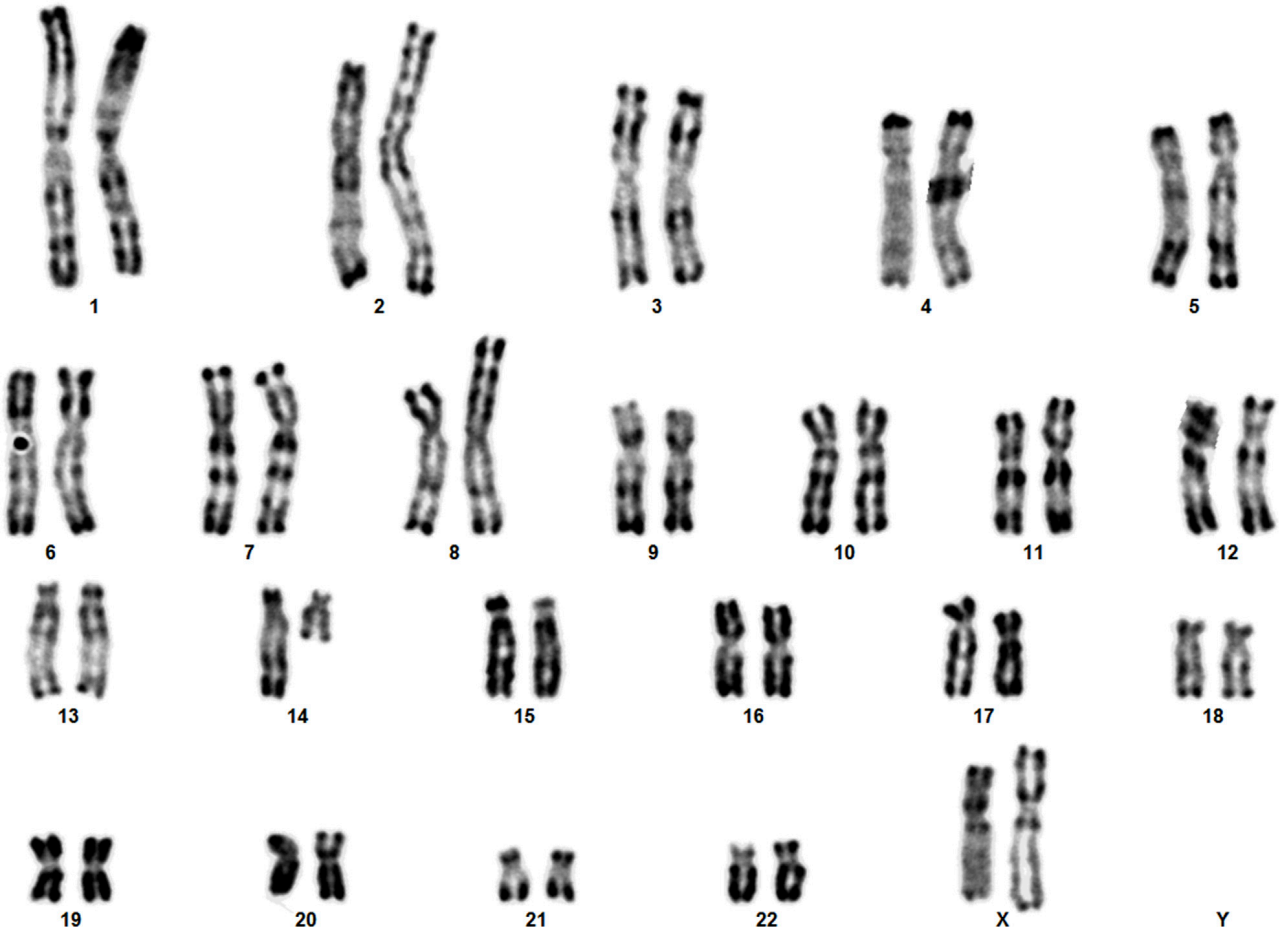
The karyotype of the mother showing the balanced translocation.

**FIGURE 4:**
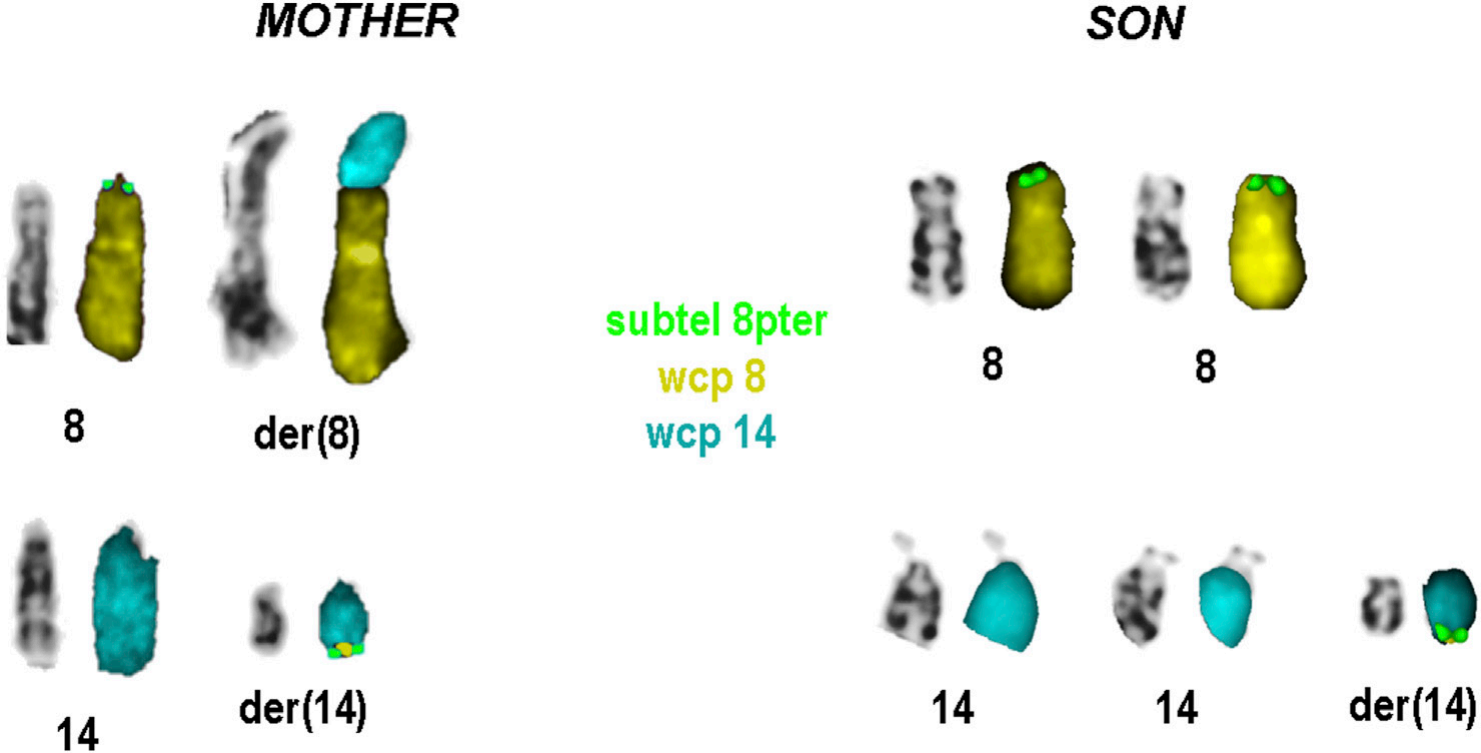
Result of molecular cytogenetics for the patient and his mother. For each (derivative) chromosome left inverted DAPI-banding, and right the FISH-results are shown.

**FIGURE 5:**
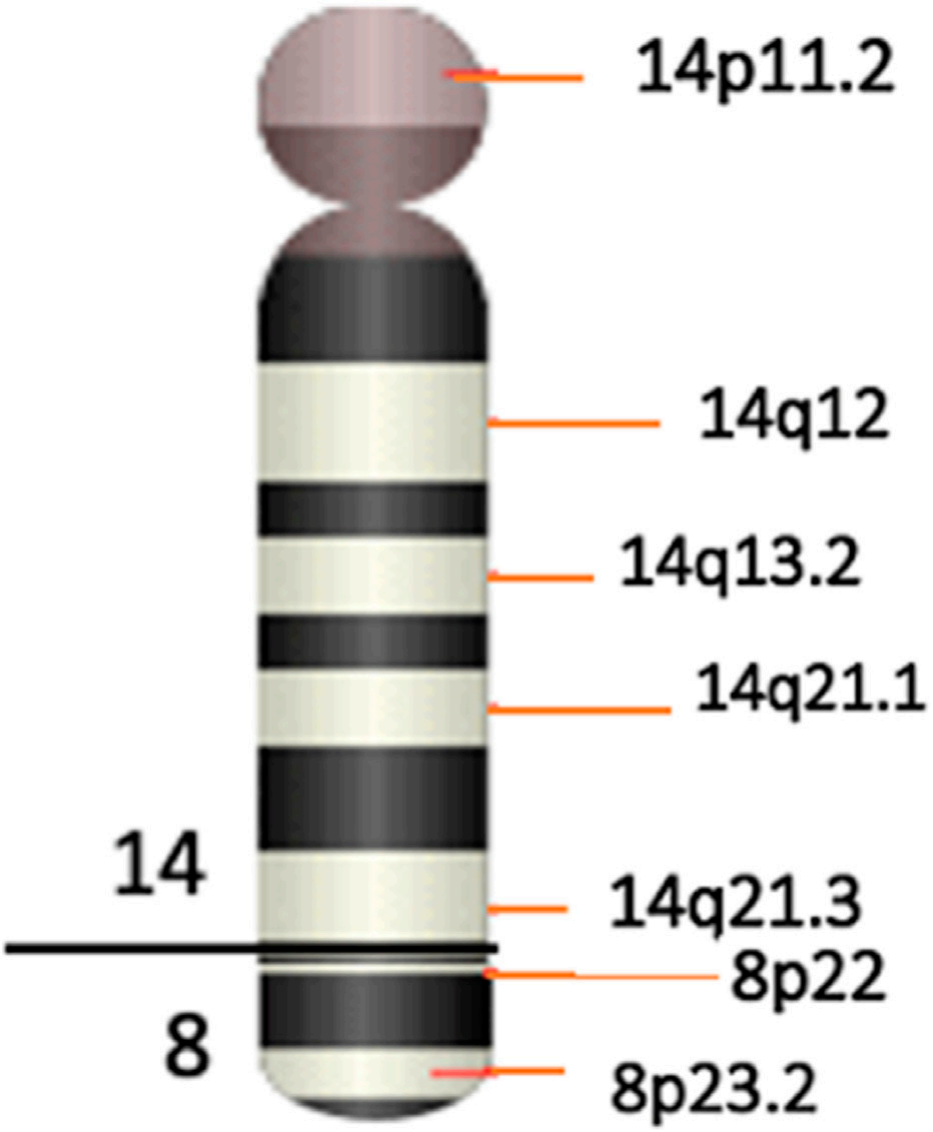
Ideogram of the sSMC(14) as a der(14)t(8;14)(p22.3;q21).

**FIGURE 6:**
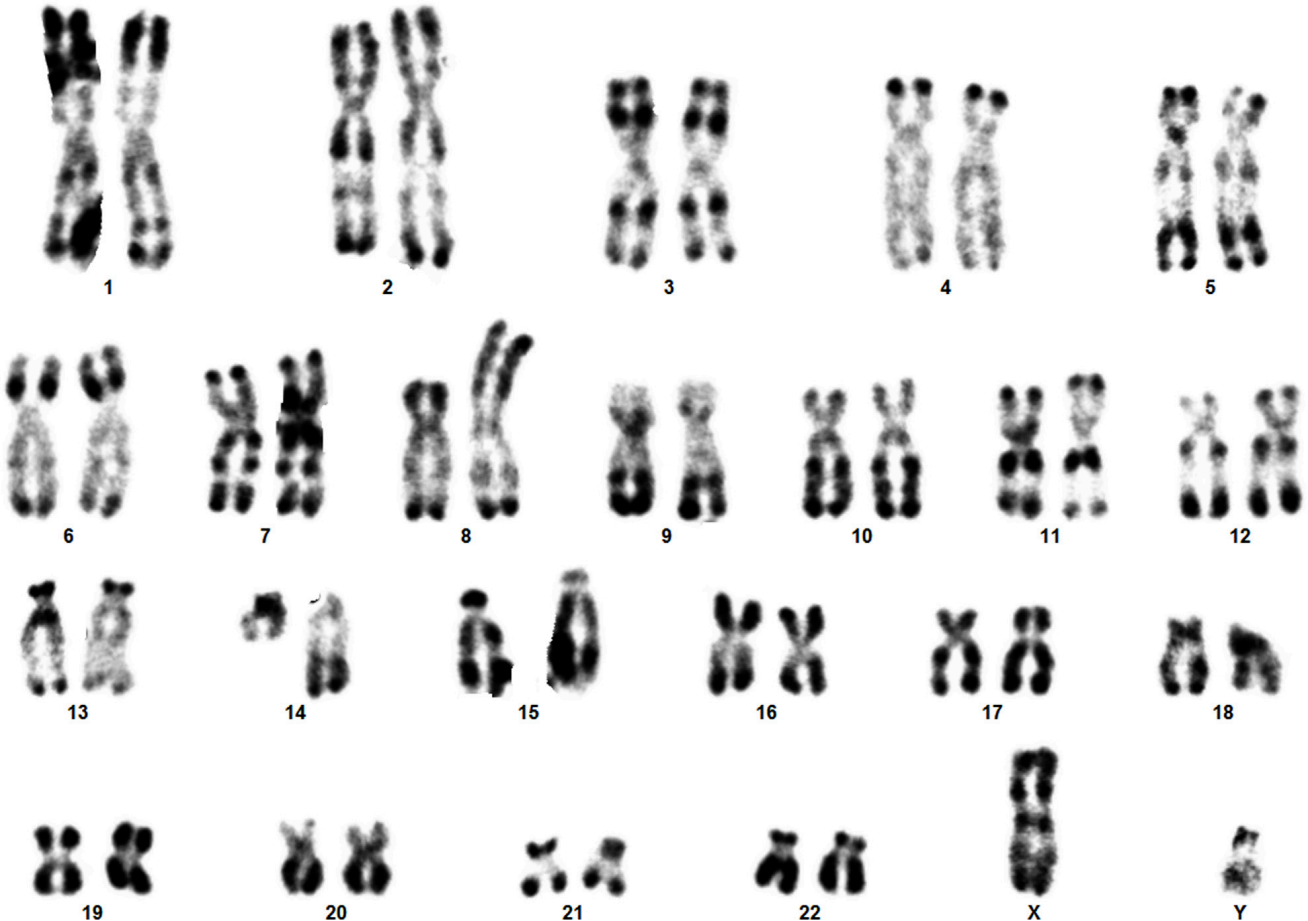
The karyotype of brother showing the balanced translocation.

Karyotype analyses showed an sSMC suspected to be derived from chromosome 14 in the patient (Figure 2) and a balanced translocation involving the chromosomes 8 and 14 in the mother (Figure 3). FISH experiments did confirm this balanced translocation in the mother classified as 46,XX,t(8;14)(p22.3;q21).ish t(8;14)(wcp14+,wcp8+,D8S504-;wcp14+,wcp8+,D8S504+) and a complex sSMC in the son, (Figure 4). Thus, this patient had a partial trisomy of the 8p22.3-8pter regionand short-arm centromere region and proximal part of the long arm of chromosome 14 (Figure 5). The molecular cytogenetic result for the patient was as follows: 47,XY,+der(14)t(8;14)(p22.3;q21)mat.ish der(14)(wcp14+,wcp8+,D8S504+) (Figure 5). After parents’ request, cytogenetic investigation of the older brother, aged 5 years and unaffected, showed that he is a carrier of the balanced maternal (Figure 6).

## Discussion

Genomic imbalances, encompassing deletions, duplications, triplications, or amplifications, have the potential to cause a spectrum of conditions including mental retardation and a range of congenital anomalies, with the specific outcomes’ contingent upon the source and type of genetic material involved. Such imbalances can also go undetected in case of being too small, and beyond the detection capability of banding cytogenetics (Liehr, 2009).

Here we have identified a rare complex sSMC originating from chromosomes 8 and 14. This case shows parallels with the first reported complex sSMC(14) described by (Guilherme et al., 2013) in 2013, which also resulted from a translocation between chromosomes 8 and 14 (Table 1). Moreover, two additional cases of complex sSMCs from translocations involving chromosomes 8 and 14 have been documented (Soudek et al., 1978; Moore et al., 1992), but the breakpoint locations differ from those in our patient, specifically at 8p and 14q [see also cases 14-Uc-10, 14-Uc-11, 14-Uc-26 (Liehr, 2023)]. There are overall 27 complex sSMCs derived from chromosome 14 (Liehr, 2023).

**TABLE 1:** Features of five patients with partial 14 trisomy.

	Faas et al. (2006)	Qi et al. (2013)	Guilherme et al. (2013)	Merhni et al. (2019)		This study
Additional chromosomal region	der(14)(pter→q21.1:) der(14)(pter→q11.2:)	der(14)(pter→q12:)	der(14)t(8;14) (p23.2;q22.1)	Case 1	Case2	der(14)t(8;14)
				der(14)(pter→q21.1:)	der(14)(pter→q21.2:)	(p22.3;q21)
Gender	M	M	M	M	M	M
Age at diagnosis	newborn	2y3m	2y9m	5y	10m	14m
Cleft lip/palate	+	+	+	_	+	+
Umbilical hernia	+	+	+	NR	NR	+
Flat nose bridge or prominent nose	NR	+	+	_	+	+
Low set ears	+	+	+	_	_	+
Microcephaly	+	_	+	+	+	+
Eye anomalies	Microphtalmia	Strabism mild ptosis	Oblique palpebral fissures	Unilateral convergent strabism	_	Microphtalmia
Hypo- or hypertelorism	_	+	+	_	_	+
Hypotonia	+	+	+	+	+	+
Craniosynostosis	+	+	_	NR	NR	+
Limb abnormalities	_	NR	Clinodactyly of the 5th fingers	Slender fingers	Fist hand Overlapping toes and fingers	Clinodactyly
Genital anomalies	_	NR	Bilateral testicular retraction	Micropenis	Micropenis	_
Growth and developmental delay	+	+	+	+	+	+
Intellectual delay	NR	+	+	+	NR	NR
Others features	Deceased in the first month due to complications arising from aspiration issues	Recurrent respiratory infections	Recurrent bronchopneumonia	Delay of tooth eruption	Pulmonary congestion	Respiratory infections
*De novo* or inherited	*de novo*	*de novo*	*de novo*	*de novo*	*de novo*	mat

Abbreviations: M, male; m, months; mat, maternal; NR, not reported; y, years

As shown in Table 1 several clinical features such as growth delay, psychomotor development delay, hypotonia, facial dysmorphia, microcephaly, umbilical hernia were found in our proband and in patients with partial trisomy 8 and 14. We suggest that the predominant clinical features observed in our patient result mainly from trisomy 14q, as they are similar to pure proximal partial trisomy 14q cases (Merhni et al., 2019) also included in Table 1. Parts of clinical variability noted between the five patients could be due to the additional abnormalities observed, like trisomy 8p. Also, in case reported by (Faas et al., 2006) in 2006 there was an additional derivative chromosome 14 in a mosaic state.

The duplication of the genomic region 8p22p23.3 has been linked to instances where individuals are clinically normal or have a mild intellectual disability without notable dysmorphic features. This suggests that the genes in this region may have a limited influence on neurobehavioral and physical development (Brooks et al., 1998).

Thereby, we assume that the predominant clinical features observed in our patient result mainly from trisomy 14q. According to Mapview (https://www.ncbi.nlm.nih.gov/mapview), the duplicated region 14q11-q21 contains several genes, including 137 identified in the OMIM database. Among these, the genes *FOXG1* (forkhead box G1), that plays a critical role in brain development. Studies have shown it to be dose-sensitive, meaning that variations in its expression levels can have significant impacts. *FOXG1* is important both in the development of the brain during prenatal stages and in postnatal neurogenesis (Hettige and Ernst, 2019). The duplication of the *FOXG1* gene has been linked to severe developmental retardation, as evidenced in various studies (Brunetti-Pierri et al., 2011). Meanwhile, the role of additional genes in the large sized involved regions cannot be excluded. Table 2 presents an overview of certain genes implicated in the symptoms exhibited by our patient.

**TABLE 2:** Candidate genes possibly being responsible for parts of phenotype from 14q11.2 to 14q21 region.

Gene	Proteine	Location	OMIM	Function
*PNP*	Purine nucleoside phosphorylase	14q11 .2	164,050	Important for normal immune response, Neurologic disorders may also be apparent in patients with immune defects
*NOVA1*	Neuro-oncological ventral antigen 1	14q12	602,157	Encodes a neuron-specific RNA-binding protein
*FOXG1*	Forkhead box G1B	14q12	164,874	Transcription repression factor which plays an important role in the establishment of the regional subdivision of the developing brain and in the development of the telencephalon
*AKAP6*	A-Kinase AnchoringProtein 6	14q12	604,691	The encoded protein is highly expressed in various brain regions and cardiac and skeletal muscle
*NPAS3*	Neuronal PAS domainprotein 3	14q13 .1	609,430	Encodes a member of the basic helix-loop-helix and PAS domain-containing family of transcription factors. May regulate genes involved in neurogenesis
*LRFN5*	leucine rich repeat and fibronectin type III domain containing 5	14q21	612,811	Encodes a protein that belongs to the leucine-rich repeat and fibronectin type III domain-containing family of proteins. A similar protein in mouse is thought to function in presynaptic differentiation

## Conclusion

As a patient with sSMC(14) was indicative to identify a maternal balanced translocation, this case is a good example for high risk of recurrence in such familial cases. Thus, chromosomal analysis of patients and their parents and other relatives (like here the brother) are essential for correct genetic counseling.

## Data Availability

The original contributions presented in the study are included in the article/Supplementary material, further inquiries can be directed to the corresponding author.
